# Trends of bloodstream infection incidence rates among patients on outpatient hemodialysis, National Healthcare Safety Network, 2012–2021

**DOI:** 10.1017/ice.2025.80

**Published:** 2026-03

**Authors:** Qunna Li, Shannon Novosad, Brian Rha, Hannah Hua, Lucy Fike, Jose Navarrete, Lu Meng, Andrea Benin, Jonathan Edwards, Jeneita Bell

**Affiliations:** 1 Division of Healthcare Quality Promotion, Centers for Disease Control and Prevention, Atlanta, GA, USA; 2 Leidos, Atlanta, GA, USA; 3 Renal Division, Department of Medicine, Emory University School of Medicine, Atlanta, GA, USA

## Abstract

**Objective:**

The purpose of the study is to analyze bloodstream infection (BSI) data reported by outpatient hemodialysis facilities to understand temporal trends, the potential impact of infection prevention practices and the COVID-19 pandemic on BSI rates.

**Methods:**

Outpatient hemodialysis facilities report BSI data to the National Healthcare Safety Network. We used interrupted time series with mixed effects negative binomial modeling to estimate the annual change of BSI rates from 2012 to 2021, using March 2020 as the COVID-19 inflection point. The model controlled for seasonal factors, vascular access types, and facility characteristics.

**Results:**

The number of facilities used for analysis increased from 5,581 in 2012 to 7,313 in 2021. Most facilities were freestanding (range: 90%–93%) and belonged to for-profit organizations (range: 85%–88%). The annual adjusted BSI rates decreased by an average of 8.90% (95% CI: −9.10 %, −8.71%) January 2012-February 2020. The annual decrease in BSI rate was not significant during March 2020-December 2021 (*P* = 0.15). There was a level drop of 32.03% (95%CI: −33.84%, −30.17%) in BSI rates in the period of March 2020-December 2021 compared with the period of January 2012-February 2020.

**Conclusions:**

BSI rates decreased steadily from January 2012 to February 2020 likely due to the identification and adoption of evidence-based prevention practices. BSI rates plateaued at lower levels during March 2020-December 2021. This suggests that infection prevention measures implemented by facilities prior to the emergence of COVID-19 contributed to substantial decreases in BSI rates and may have helped to stabilize BSI rates after March 2020.

## Introduction

From 2012 to 2020, persons in the United States with end-stage renal disease (ESRD) increased from approximately 600,000 to 800,000.^1^ In 2020, more than 60% received hemodialysis at one of the more than 7,000 outpatient dialysis facilities. Infections are a leading cause of hospitalization and death among ESRD patients who receive hemodialysis treatments. These patients are at risk of infection because of repeated use of vascular access sites, frequent hospitalizations, and immune dysfunction.^1–4^

Bloodstream infections (BSI) are one of the most common types of infections in patients on hemodialysis. The type of hemodialysis vascular access is an important risk factor for BSIs, with central venous catheters (CVC) having the highest risk, followed by arteriovenous grafts (AVG) and arteriovenous fistulas (AVF).^5–7^ Other factors, such as seasonality, have also been associated with increased BSIs.^8–11^

CDC’s National Healthcare Safety Network (NHSN) has been collecting hemodialysis event data, including BSIs, since 2005. These data have been used by the Centers for Medicare and Medicaid Services (CMS) as part of ESRD Quality Incentive Program (QIP) since 2012, and resulted in near universal reporting from outpatient hemodialysis facilities across the United States. BSI standardized infection ratios (SIR) developed by NHSN decreased from 1.00 in 2014 to 0.60 in 2019.^6^ However, a detailed study of this decline has not been done.

In addition to BSI risk, patients on outpatient hemodialysis suffered substantial morbidity and mortality related to COVID-19.^1^ Dialysis facilities quickly implemented heightened infection prevention and control (IPC) measures to provide lifesaving therapies for these highly vulnerable patients in the presence of severe staffing shortages.^12^ Personal protective equipment (PPE) and other supply shortages early in the pandemic further stressed facilities. Some studies have described decreased BSIs during the COVID-19 pandemic in dialysis settings, in contrast to other healthcare settings.^13–16^ However, BSI rates in dialysis facilities during the COVID-19 pandemic on a nationwide scale have not been described.

The purpose of the study is to analyze national dialysis BSI surveillance data reported to NHSN from 2012-2021 and examine the trend of BSI incidence rates before and after March 2020, controlling for seasonal patterns, vascular access type, and facility characteristics.

## Methods

### Data source and definitions

We analyzed BSI data reported to NHSN from 2012-2021 using interrupted time series, with March 2020 as the COVID-19 interruption. Outpatient hemodialysis facilities participating in NHSN are required to follow a standard surveillance protocol developed by NHSN.

BSI events were defined as positive blood cultures that were collected in an outpatient setting or collected within one calendar day after hospital admission. There must be 21 or more days between positive blood cultures for each positive blood culture to be considered a separate event. During the selected study period of 2012–2021, BSI events were reported using a standard data collection form that includes vascular access in place at the time of the BSI event, patient demographic data, associated clinical symptoms, and event-related outcomes.

Participating facilities also reported the number of outpatients who received hemodialysis during the first two working days of each month, stratified by hemodialysis vascular access type. Hemodialysis vascular access types, in the order of decreasing risk of infection, include non-tunneled CVC, tunneled CVC, other vascular access device (eg, catheter-graft hybrid, port), AVG, and AVF. If the patient had more than one vascular access types, only the vascular access type with the highest risk of infection was entered.

Additionally, facilities are required to complete an annual survey that collects data on facility characteristics and infection prevention practices.

### Statistical analyses

Events were excluded (<0.2% of events reported) if they appeared to be duplicates, violated the 21-day rule, missing vascular access type or patient-months data. The number of BSI events and number of outpatient dialysis patients for each facility-month were summarized by vascular access type. If a patient had more than one vascular access type associated with a BSI event, the event was attributed to the highest risk access using the risk scale described above.

To investigate the trend of BSI incidence rates and account for any changes associated with the COVID-19 pandemic, we used interrupted time series with mixed-effects negative binomial model (ITS). The dependent variable was the monthly facility-level count of BSI events by access type with the natural logarithm of the facility’s monthly number of hemodialysis outpatients as the offset. The main independent variables of interest were a linear term for time in month, a binary indicator variable for before or after March 2020 (the widely accepted start of the COVID-19 pandemic in the U.S.), and an interaction term between the binary indicator variable and a linear term in month since March 2020. Annual change in BSI incidence rates was estimated before the COVID-19 period (January 2012–February 2020), a level-change of BSI incidence rates on and after March 2020, and the annual change of BSI incidence rates during the COVID-19 period (March 2020–December 2021). We also introduced a sine and a cosine function pair of one cycle per 12 months (sine=sin(2*π*month/12; cosine=cos(2*π*month/12)) to assess and control for seasonality. The parameter estimates of the sine and cosine function were used to identify the months with the highest and lowest BSI rates, and their rate ratio.^17^ Other covariates included vascular access type and facility characteristics such as hospital affiliation, member of a group or chain of dialysis centers, number of in-center hemodialysis stations, and ownership of the dialysis center. To evaluate sample migration of facilities reporting over time, sensitivity analyses were conducted using continuous reporters defined as reporting ≥6 months of data each year between 2013 and 2021. We re-fit the model to see if the results had changed. Data for 2012 was excluded from sensitivity analysis because the QIP program was implemented in 2012 and many facilities started annual reporting in 2013 to NHSN. Analyses were conducted using SAS software (version 9.4; SAS Institute).

## Results

### Facility characteristics

The number of outpatient hemodialysis facilities reporting to NHSN increased from 5,581 in 2012 to 7,313 in 2021. Most facilities were freestanding (range: 90%–93%), for-profit organizations (range: 85%–88%), and part of a dialysis corporate chain (range: 89%–93%). The median number of stations remained 17 (interquartile range, 12–24) over the study period (Table 1).

**Table 1.** Characteristics of outpatient hemodialysis facilities reporting to the national healthcare safety network (NHSN), 2012 – 2021

Characteristic	
**No. of facilities reporting, range over the years** ^*^	5581 – 7313
**For-profit ownership, %, range over the years** ^*^	85 – 88
**Part of a dialysis corporate chain, %, range over the years** ^*^	89 –93
**Freestanding clinic (not hospital owned/affiliated), %, range over the years** ^*^	90 – 93
**Number of stations, median (IQR)** ^**^	17 (12 –24)

* *Values varied from year to year, so a range was provided to describe range of values between different years.*

** *IQR = interquartile range, the median and IQR for number of stations remained the same over the years, so one single median and IQR value was provided for number of stations.*

### Crude BSI rates

The yearly crude BSI incidence rates for all access types decreased steadily from 2012 until 2020. Patients with CVCs, AVGs, and AVFs had BSI rates of 2.75, 0.41, and 0.29 per 100 patient-months in 2012, which decreased to 0.80, 0.21, and 0.12 per 100 patient-months, respectively, in 2020. However, the crude rates in 2021 appeared similar to the crude rates in 2020 for all vascular access types. From January 2012 to February 2020, the pooled crude BSI rates were 1.82, 0.36, and 0.22 per 100 patient-months, respectively, when stratified by CVCs, AVGs, and AVFs access type. During March 2020-December 2021, pooled crude BSI rates were 0.79, 0.21, and 0.11 per 100 patient-months, respectively, when stratified by CVCs, AVGs, and AVFs (Table 2).

**Table 2.**
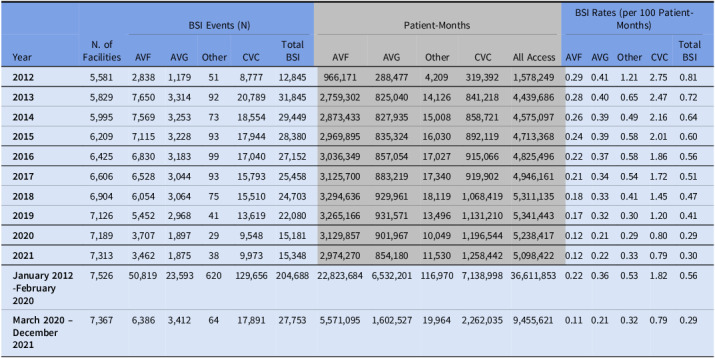
Yearly crude bloodstream infection (BSI) rates among outpatient hemodialysis patients by vascular access type, 2012 – 2021

***Abbreviations:** AVF = arteriovenous fistulas; AVG = arteriovenous grafts; CVC = central venous catheters; BSI = bloodstream infection.*

### ITS trends

**Figure 1.**
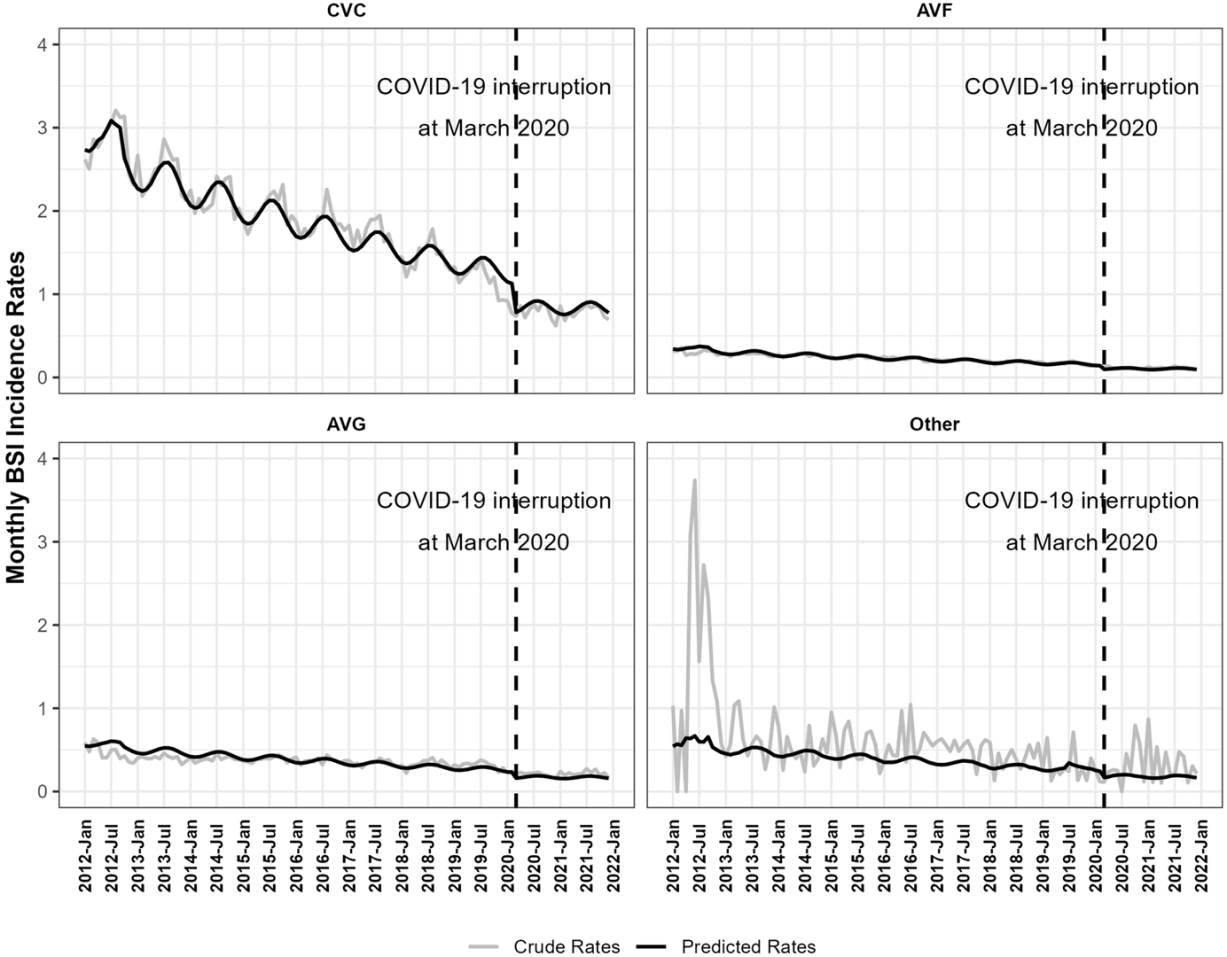
Monthly crude and predicted bloodstream infection (BSI) per 100 Patient-Months by Vascular Access Type, NHSN, 2012 - 2021. Abbreviations: AVF, arteriovenous fistulas; AVG, arteriovenous grafts; CVC, central venous catheters. COVID-19 interruption at March 2020: this study defined March 2020 as the start of COVID-19 pandemic in the U.S.

ITS model adjusted for vascular access types, location/hospital affiliation, and seasonal factors. The adjusted BSI rates decreased by an average of 8.90% (95%CI: −9.10%, −8.71%) annually from January 2012 to February 2020. There was a level drop of 32.03% (95%CI: −33.84%, −30.17%) in BSI incidence rates in the period of March 2020-December 2021 compared with the period of January 2012-February 2020. The change in slope during March 2020-December 2021 was 7.94% (95%CI: 5.47%, 10.47%) compared with January 2012-February 2020 period. As a result, the decrease in BSI rates from March 2020 to December 2021 was not significant at 1.68% (95%CI: −3.92%, 0.61%; p-value = 0.15) annually (Table 3). Monthly predicted BSI rates by access type from ITS model were shown in Figure 1.

**Table 3.** Bloodstream infection (BSI) interrupted time series model estimated incidence rate ratios and annual percentage change

Model Parameter^a^	Parameter Estimates at monthly level	Yearly aRR (95% CI)	Percent change per year^b^ (95% CI)	p-value
**Time Trend before COVID-19, JAN2012-FEB2020 (β1)** ^c^	−0.008	0.91 (0.91, 0.91)	−8.90 (−9.10, -8.71)	<.001
**Change in slope during COVID-19 period, MAR2020 - DEC2021 (β2)** ^c^	0.006	1.08 (1.05, 1.10)	7.94 (5.47, 10.47)	<.001
**Time Trend during COVID-19, MAR2020-DEC2021 (β1+ β2)** ^c^	−0.001	0.98 (0.96, 1.01)	−1.68 (−3.92, 0.61)	0.15
	**Parameter Estimates**	**aRR (95% CI)**	**Percent change** ^b^	**p-value**
**Level change of COVID-19 interruption at March 2020**	−0.386	0.68 (0.66, 0.70)	−32.03 (−33.84, -30.17)	<.001
**Vascular access types**				
CVC	2.090	8.09 (8.01, 8.17)	–	<.001
AVG	0.495	1.64 (1.62, 1.67)	–	<.001
Other	0.489	1.63 (1.49, 1.78)	–	<.001
AVF	Ref	Ref	–	–
**Location/Hospital affiliation**				
Hospital^d^	0.155	1.17 (1.14, 1.20)	–	<.001
Freestanding	Ref	Ref	–	–
**Sine function**	−0.077	–	–	<.001
**Cosine function**	−0.056	–	–	<.001

***Abbreviations**: AVF = arteriovenous fistulas; AVG = arteriovenous grafts; CVC = central venous catheters;* *a RR = adjusted rate ratio.*

a Negative binomial mixed model adjusted for vascular access type, seasonal factors (sine: sin(2*π*month/12 and cosine: cos(2*π*month/12)) and location/hospital affiliation.

b *Percent change=(*^a^*RR-1)×100.*

c *Data modeled at facility-month level, aRR and Percent change calculated at yearly level.*

d *Location could be a hospital or a freestanding location owned by/affiliated with a hospital.*

Vascular access type was associated with BSI incidence rates. The adjusted BSI rates were 8.09 times as high for CVC vs AVF (95%CI: 8.01, 8.17), AVG vs AVF were 1.64 (95%CI: 1.62, 1.67) and other vascular access vs AVF were 1.63 (95%CI: 1.49, 1.78).

The BSI rates showed seasonal patterns. The model indicated on average the BSI rates had a peak around August, and a trough around February (Figure 1). Within a year, the BSI rate ratio between the peak and trough was 1.21 (95%CI: 1.19, 1.23).

### Sensitivity analysis

There were 5,247 (68%) facilities that continually reported ≥6 months of data each year for 2013–2021. The adjusted BSI incidence rates decreased by an average of 8.85% (95%CI: −9.07%, −8.63%) annually from January 2013 to February 2020 for those continuous reporters. There was a level drop of 31.70 % (95%CI: −33.70%, −29.64%) in BSI incidence in the period of March 2020-December 2021 compared with the period of January 2013-February 2020. The decrease in BSI incidence rates was not significant during March 2020-December 2021 at 1.50% (95%CI: −3.97%, 1.04%) annually.

## Discussion

### Key findings

The adjusted rate of BSI in patients receiving outpatient hemodialysis decreased by an average of 8.90% annually from January 2012 to February 2020. Adjusted rates were lower in March 2020–December 2021 [32.03% level drop] compared to the January 2012–February 2020 period and the change of BSI rates became insignificant from March 2020 to December 2021. The BSI incidence rates showed clear seasonality, with a peak around August and a trough around February.

### Comparison with other studies

The marked reductions we observed in BSI incidence rates in patients receiving outpatient hemodialysis during 2012–2020 can likely be attributed at least in part to major practice changes in the field including widespread uptake of evidence-based interventions proved to reduce BSIs and advances in technology. Data is not available on the uptake and implementation of specific practices and products used at each dialysis facility, so the impact of individual interventions during this time period is difficult to describe. It is likely multifactorial with the impact of different practices varying at each facility.

In 2009, CDC launched a dialysis BSI prevention collaborative to determine whether BSI rates in outpatient hemodialysis centers could be reduced with improved adherence to recommended IPC practices (ie, the Core Interventions). The intervention bundle included, among other practices, surveillance of BSI through NHSN, CVC care practices, and staff competency observations. These practices showed a 54% decrease in access-related BSIs during the intervention period. A follow-up analysis confirmed that the reduction was sustained for at least four years after the intervention and was most pronounced in patients with CVCs.^18,19^ A large cluster-randomized trial conducted by a large dialysis organization complemented this work and showed that implementing two CDC-recommended catheter care practices (using chlorhexidine with alcohol for exit site care and scrubbing the CVC hub with an antiseptic) led to a 41% reduction in BSIs in patients with catheters over one year.^9,20^ In 2016, CDC launched the Making Dialysis Safer for Patients Coalition, a partnership among a broad range of healthcare, public health, and patient safety organizations and individual stakeholders with a mission to prevent BSIs and promote the Core Interventions.^21^ While the implementation of the Core Interventions in dialysis facilities across the U.S. is difficult to measure, work by this coalition and others in the kidney community to encourage the adoption of IPC practices has likely contributed to the decline in BSI rates.

Apart from the Core Interventions, other advances occurred in IPC practices during this time period. For example, studies performed by large dialysis organizations have shown that antiseptic-impregnated caps are associated with decreases in BSIs. Specifically, in one study, the use of caps with chlorhexidine-impregnated rods (ClearGuard HD) compared to standard CVC caps decreased BSIs by 56%. While exact data are not available on the routine use of these caps, at least one large dialysis organization began using them in their facilities nationwide in 2019.^22^ Given these products were not available and/or commonly used in dialysis facilities until the later years of the analysis, they might have influenced later decreases. In addition, dialyzer reuse, which has been associated with gram-negative BSIs, has decreased dramatically over this time in U.S. dialysis facilities. In 2012, approximately 24% of facilities reported reuse (down from a peak of >80% in the late 1990s), this number has steadily decreased in 2012-2021 and is now very rare in the U.S.^23–25^

March 2020 was recognized as the start of the COVID-19 pandemic in the U.S.^26^ and used as the interruption point in the model to account for the COVID-19 pandemic in this study. This model demonstrated an adjusted BSI rate level decrease of 32% during March 2020-December 2021 compared with BSI rates in the period of January 2012-February 2020. Similarly, there have been reports in the literature of decreasing dialysis-related BSI rates after the onset of the COVID-19 pandemic. An observational retrospective study conducted on 71 chronic hemodialysis patients in two hospitals in Italy showed a 91% reduction in catheter-related BSI from February to May 2020 compared to the same period in 2019.^27^ Another study using United States Renal Data System (USRDS) data found that hospitalization rates for catheter-associated BSIs decreased between 17% and 24% during the first six months of the pandemic compared to the corresponding periods in 2019. The study also found a substantial reduction in rates of BSI in late 2019 and early 2020, which the authors attributed at least in part to the introduction of antimicrobial barrier catheter caps into routine use at one large dialysis organization.^14^ These findings contrast with what other healthcare settings experienced during the COVID-19 pandemic. A study using NHSN data reported that the national SIR of central-line-associated BSIs in acute care hospitals increased from April to June 2020 compared to the same period in 2019.^15^

We were not able to elucidate the exact causes of the level drop of the BSI rates seen in this study, nor for the observation that monthly adjusted BSI rates did not vary significantly throughout the March 2020-December 2021 period. However, these findings could be related to changes in care patterns or infection control practices. For example, COVID-19 mitigation measures and pandemic-related changes in infection prevention practices—such as increased use of PPE for both staff and patients and increased attention to environmental cleaning and disinfection practices for dialysis machines and stations—may have reduced BSI risk more successfully than the routine infection prevention measures in place prior to COVID-19 pandemic. Conversely, staffing shortages, supply shortages, and other challenges (eg, decreased patient attention to vascular site care at home) may have lessened this potential impact on BSIs at a nationwide level, possibly leading to a plateau in monthly BSI rates.

Furthermore, changes in the number of patients with specific types of vascular access may play a role. According to the 2022 USRDS annual report, the percentage of patients initiating hemodialysis with a catheter increased by 1.5% between 2019 and 2020, and the percentage started with an AVF decreased by 1.1%; the percentage of patients with a catheter in use for at least three months increased by 20%.^1^ As supported by our study and others, hemodialysis patients with CVC have the highest risk of BSIs, while patients with AVF have the lowest risk. Longer than three months of catheter use also plays a role in increased BSI risk.^5–7,28^ Although any of the above factors may have contributed to the difference in BSI rates observed during the March 2020–December 2021 period, we were not able to directly assess their impact in this analysis.

The observed seasonal pattern of BSI incidence rates in our study is consistent with what has been reported in the literature.^8,9^ In summer months, when the temperature and humidity are higher, it may be difficult to maintain the protective barriers needed to keep the catheter exit site clean and intact. Further, the summer environment may enhance the growth of bacteria and fomites. In a clustered randomized quality improvement initiative study, the seasonality of ‘summer bloom’ of bacteremia was observed in the control facilities, but the seasonal trend of the summer months was attenuated in the intervention facilities where the use of 2% chlorhexidine with 70% alcohol swab sticks for exit site care and 70% alcohol pads to perform ‘scrub the hubs’ were incorporated in dialysis-related CVC care procedures, highlighting that increased emphasis on practices in the summer months may be needed.^9^

While considerable progress has been made in preventing BSIs, in 2021, a total of 15,348 BSIs still occurred, along with increased patient-months and BSI events associated with CVC access type (Table 2). Continued and renewed efforts are needed to ensure any barriers to accessing care/vascular access procedures are addressed using multidisciplinary teams. Further infection prevention work applied equitably among all facilities is needed, and barriers to implementing these practices should be explored. For example, human factors studies have shown that the fast-paced environment in a typical dialysis facility might present challenges for staff to adhere to best practices.^29^

### Strengths and limitations

This study has several limitations. First, surveillance data were used and we depend on facilities reporting data by following a standard protocol. To improve data quality, NHSN implemented business rules to limit erroneous data entry; monthly data quality checks are employed and quarterly data validation on random samples are conducted by NHSN staff. Second, not every facility consistently reported all data from 2012 to 2021. However, the sensitivity analysis confirmed minimum impact of unbalanced reporting on our analysis. Third, on March 22, 2020, CMS announced reporting exceptions for data from October 1, 2019 to June 30, 2020 for QIP facilities due to COVID-19. As a result, data for January 2020-June 2020 may have been impacted; however, the number of facilities and total patients for those months are comparable to ensuing months, which suggests that most facilities still reported data to NHSN regardless of the reporting exceptions. Finally, although March 2020 was recognized as the start of the COVID-19 pandemic in the U.S. and used as the interruption point in the study, the exact timing of the impact of the COVID-19 pandemic may vary in different parts of the country.

These findings suggest that infection prevention measures implemented by facilities prior to the emergence of COVID-19 might have contributed to steady reductions in BSIs before the pandemic and mitigated challenges experienced by patients and facilities during the pandemic.
